# Factors influencing nurses’ intention to care for patients with COVID-19: Focusing on positive psychological capital and nursing professionalism

**DOI:** 10.1371/journal.pone.0262786

**Published:** 2022-01-19

**Authors:** Sun-a Jeong, Jinhee Kim

**Affiliations:** 1 Department of Nursing, Chosun University Hospital, Gwangju, South Korea; 2 Department of Nursing, College of Medicine, Chosun University, Gwangju, South Korea; Murcia University, Spain, SPAIN

## Abstract

**Purpose:**

It is necessary to identify factors that influence nurses’ intention to care for coronavirus disease 2019 (COVID-19) patients to improve the quality of care during the pandemic. This study identifies factors that influence nurses’ intention to care for COVID-19 patients, focusing on positive psychological capital and nursing professionalism.

**Methods:**

This study adopted a descriptive correlational design. Data were collected between August 16 and August 30, 2020, through self-administered questionnaires from 148 bedside nurses caring for COVID-19 patients, from four hospitals designated for COVID-19 treatment. Modified versions of the Nursing Intention Questionnaire for SARS Patient Care, Psychological Capital Questionnaire, and Hall’s Professional Inventory were used. The collected data were analyzed using stepwise multiple regression.

**Results:**

In total, 165 questionnaires were distributed, and 148 questionnaires (89.7%) were included in the final analysis. Factors influencing nurses’ intention to care were: age (30<: β = .18, *p* = .026; ≥50: β = .23, *p* = .005), department (ICU: β = -.26, *p* = .001), sufficient clinical experience and skills to care for COVID-19 patients (sufficient: β = .18, *p* = .019), and positive psychological capital (β = .22, *p* = .044). The model’s explanatory power (R^2^) was 48%.

**Conclusions:**

Strategies to increase nurses’ positive psychological capital are necessary to improve nursing care quality by increasing intention to care when facing novel infectious diseases such as COVID-19. Furthermore, adequate education and training on managing novel infectious diseases should be implemented to provide nurses with relevant experience and skills regarding caring for patients infected with these diseases. Through various studies, strategies for improving nurses’ positive psychological capital need to be suggested to improve the quality of care by increasing the nurses’ intention to care during the emergence of a novel infectious disease, such as COVID-19. Additionally, adequate education and training on managing the novel infectious diseases, sufficient for the nurses to believe they have the experience and skills for caring for the infected patients, will be needed.

## Introduction

Following the first case of COVID-19 in Wuhan, Hubei Province, China in December 2019, the first confirmed case in South Korea was reported in January 2020 [1]. COVID-19 spread worldwide rapidly, and in March 2020, the World Health Organization (WHO) officially declared it a pandemic [2]. Currently, approximately 176,532,000 confirmed cases and 3,819,000 deaths have been reported worldwide [3], while in Korea, approximately 150,000 confirmed cases (cumulative) and 2,000 deaths have been reported [4]. Although most countries have implemented strict social distancing measures such as school closures and restricting gatherings, the dissemination rate of the virus has not significantly decreased [3, 4].

COVID-19 patients require care from clinicians including nurses. Among clinicians, nurses are particularly affected by COVID-19; they have to provide direct bedside care including medication administration, sample collection, administration of intravenous injections, ventilator management, and monitoring patient status while wearing personal protective equipment [5]. Most nurses were unprepared for the duties assigned to them with respect to nursing care of COVID-19 patients; as a result, they have been suffering from work burnout due to the burden of work as well as anxiety, depression, and fear [5, 6]. Such negative work experiences have decreased nurses’ intention to care, such as their refusal to care for patients and turnover intention [5, 7]. Thus, it is necessary to identify factors that positively influence nurses’ intention to care and develop strategies that strengthen them.

Intention to care refers to nurses’ willingness to care for patients voluntarily [8]. In the current crisis situation, identifying the factors influencing nurses’ intention to care is essential not only to reduce the levels of fear, anxiety, stress, and turnover intention among nurses, but also to improve the quality of nursing care [9].

Additionally, in the current COVID-19 pandemic, nurses are experiencing fear, anxiety, depression, helplessness, and exhaustion stemming from the fear of getting infected themselves or spreading the virus to their families [5, 6, 10]; all of these have a negative impact on individuals’ sense of optimism [11]. Reduced optimism can increase fear of COVID-19, leading to a vicious cycle [11]. As such, nurses should learn to utilize self-coping mechanisms that involve psychological and lifestyle adjustments [10]. Although nurses who care for COVID-19 patients may initially experience negative emotions, over time, their psychological defense mechanisms enable positive emotions to co-exist [5, 10]. Therefore, strategies to increase nurses’ optimism need to be developed.

Positive psychological capital is a concept derived from an organizational application of positive psychology—organizational behavior research and evolving topics—and refers to the complex positive psychological state of members in an organization. It is a second-order factor with a multidimensional construct, and is composed of the sub-domains of self-efficacy, hope, optimism, and resilience [12]. Although previous studies reported a negative correlation between positive psychological capital and exhaustion and turnover intention [13], studies in context of nursing are still lacking. Furthermore, research on positive psychological capital in the context of crises such as the COVID-19 pandemic are yet to be conducted. Thus, the positive psychological capital of nurses should be explored to better understand their intention to care during the COVID-19 pandemic.

The role of a nursing professional with a positive professional outlook is critical for an effective response to unprecedented disaster situations such as the spread of a novel infectious disease [14]. High-quality care and efficient performance is possible when nurses have a positive and clear sense of professionalism [15]. Certainly, nurses’ intention to care was low during the SARS and Ebola epidemics [7, 16]. Although nursing professionalism has been suggested as a factor that positively influences nurses’ intention to care for patients in the pandemic situation [17], verification is needed as some studies have reported no relevance in this regard [9, 18].

As crises caused by a novel infectious disease outbreak can recur in the future, improving the quality of nursing by identifying factors that influence nurses’ intention to care during these situations is imperative. This study aims to examine nurses’ intention to care for COVID-19 patients in the prevailing pandemic situation, and to identify factors that influence their intention to care. The current study focuses on positive psychological capital and nursing professionalism to provide preliminary data to support efforts to positively influence nurses’ intention to care.

## Methods

### Study design

This study used a descriptive correlational investigation to examine the level of intention to care for patients among bedside nurses caring for COVID-19 patients. The study also aimed to identify factors that influence their intention to care, based on the concepts of positive psychological capital and nursing professionalism.

### Participants and data collection

The participants of this study were nurses working at one of the two university hospitals with nationally designated negative pressure isolation rooms, or two national hospitals designated as treatment facilities for patients with COVID-19. Participants had to satisfy the following conditions: first, they had to be nurses who directly cared for COVID-19 patients; and second, they had to provide informed consent and voluntarily agree to participate therein.

The sample size for this study was calculated using the G*power 3.1.9.4 program following previous research [18] on factors influencing nurses’ intention to care. The minimum required number of participants was 147, considering a two-tailed test for multiple regression, significance level α = .05, statistical power (1-β) = .90, moderate effect size (f^2^) = .15, and 10 predictors included in the regression analysis. Ultimately, 165 nurses were included in the study to account for attrition.

Data were collected between August 16 and 30, 2020, from two university hospitals with nationally designated negative pressure isolation rooms and two national hospitals designated as COVID-19 treatment centers. Prior to data collection, the nursing department at each hospital was effectively conveyed the purpose of the study and requested to cooperate, via telephone. Approval to conduct the study was also obtained prior to data collection. Although the investigator hoped to collect data through in-person visits to the three hospitals, with the exception of the investigator’s own workplace, the hospitals informed that in-person visits were not possible during the COVID-19 pandemic. Therefore, the investigator provided the description, consent form, questionnaires, and return envelopes to the nurse-in-charge of the COVID-19 patients at each hospital, for distribution among the participants. Study participants then filled out the consent forms and the questionnaires and returned them to the nurse-in-charge in a sealed return envelope. The nurse-in-charge collected and boxed these documents and mailed them to the investigator. At the hospital where the investigator is employed, the investigator delivered the aforementioned documents to the nurse-in-charge for data collection, and the latter distributed these documents to the participants. Signed consent forms and completed questionnaires were returned to the nurse-in-charge in the return envelope, which were then collected by the investigator.

In total, 165 questionnaires were distributed, of which 156 were collected, indicating a recovery rate of 94.5%. Of these, 8 questionnaires were deemed unsuitable for statistical analysis due to inappropriate responses; these were excluded. Finally, 148 questionnaires were used in the final analysis, indicating a response rate of 89.7%.

### Questionnaires and measurements

#### Intention to care

Intention to care was measured using a tool developed by Yoo et al. [19]; it assessed nurses’ intention to care for severe acute respiratory syndrome (SARS) patients. The instrument was then modified and revised by Lee and Kang [9] to ensure its validity for use on nurses caring for patients with novel infectious diseases. Permission to use the revised version was obtained from Lee. The tool consisted of three items measured on a seven-point Likert scale ranging from “strongly disagree” (-3 points) to “strongly agree” (3 points). The final score was calculated by obtaining the average of the scores of the three items. A higher average score indicated greater intention to care. The reliability of the tool according to Lee and Kang [9] was Cronbach’s α = .88, and in this study, Cronbach’s α = .97.

#### Positive psychological capital

Positive psychological capital was measured using the Psychological Capital Questionnaire (PCQ) developed by Luthans et al. [12], translated to Korean and tested for validity by Lee and Choi [20]; it was then revised by Lee [21] to fit the nursing context. Prior to using the tool, approval was obtained from the Mind Garden, Inc. (www.mindgarden.com)—which owns the PCQ copyright—and the Korean translators and editors. The tool comprises 24 items in 4 subdomains: self-efficacy (6 items), optimism (6 items), hope (6 items), and resilience (6 items) measured on a six-point Likert scale ranging from “strongly disagree” (1 point) to “strongly agree” (6 points). The score was obtained by calculating the average of the scores on the 24 items. Higher scores indicated higher levels of positive psychological capital. The reliability of this tool was reported as Cronbach’s α = .88–.89 in a study by Luthans et al. [12], while Lee and Choi [20] reported Cronbach’s α = .93. Similarly, Lee [21] reported a reliability of Cronbach’s α = .95. In this study, Cronbach’s α = .94.

#### Nursing professionalism

Nursing professionalism was measured using Hall’s Professional Inventory, an assessment tool developed by Hall [22], revised by Snizek [23], and translated into Korean and validated by Baek and Kim [24]. Prior approval for use was obtained from Baek. The tool comprises 25 items in 5 subdomains: standard of professional organization (5 items), belief in public service (5 items), autonomy (5 items), belief in self-regulation (5 items), and vocational consciousness (5 items). The scores are measured on a five-point Likert scale ranging from “strongly disagree” (1 point) to “strongly agree” (5 points). The score was obtained by calculating the average of the scores on the 25 items. Higher scores indicated higher levels of nursing professionalism. When the tool was developed, its reliability was Cronbach’s α = .86 [22], while Snizek [23] reported a reliability of Cronbach’s α = .78 and Baek and Kim [24] reported Cronbach’s α = .82. In this study, Cronbach’s α = .63.

### Ethical consideration

Approval from the Institutional Review Board (IRB) was obtained (IRB File No. CHOSUN 2020-07-007) in addition to permission obtained from the nursing director of the institutions included in the study and the cooperation of pertinent individuals. An explanation regarding the purpose of the study, voluntary participation, freedom to withdraw from or discontinue the study without penalty, anonymity, and the storage and destruction of data were provided to participants, and their written consent was obtained prior to completing the questionnaire. The questionnaire took approximately 15 minutes to complete, and participants received a small incentive (approximately $5) as a token of appreciation for their participation. The collected written consent forms were stored in a locked cabinet and the data were encrypted and computerized with a password to ensure the confidentiality of personal information. All documents and data collected in this study will be stored for three years following the completion of the study, and then discarded.

### Data analysis

Data analysis was performed using the SPSS/WIN 26.0 program according to the procedure described in this section. First, the differences between participants’ level of intention to care based on general and clinical experience characteristics were analyzed using an independent t-test or one-way ANOVA, along with the Scheffé test for the post-hoc analysis. Second, Pearson’s correlation coefficient was calculated to examine the relationship among positive psychological capital, nursing professionalism, and intention to care. Finally, a stepwise multiple regression analysis was performed using the stepwise selection method to examine the influence of positive psychological capital and nursing professionalism on nurses’ intention to care for COVID-19 patients.

## Results

### Participants’ general characteristics and characteristics of their clinical experience

The response rate for the questionnaire was 89.7%. Of the participants, 94.6% were women. Most participants were aged less than 30 years (51.4%), followed by individuals in their 30s (25.0%) and 40s (15.5%). The average age of participants was 32.67±8.60 years. Among the participants, 65% were not religious and 65.5% were not married. Most participants lived together with family (88.6%) and did not have children (69.6%). Most participants had a bachelor’s degree (63.5%). The most common duration of job experience as a nurse was more than 7 years (45.3%), followed by 1–3 years (31.8%). Only 6.1% had worked as a nurse for less than a year. The most prevalent job position was a general bedside nurse (86.5%), and the most common type of work was a three 8-hour shift pattern (91.2%). The most common subjective health status was moderate (45.9%) followed by healthy (45.3%), and unhealthy (8.8%) (Table 1).

**Table 1:** Participants’ characteristics and differences in intention to care by their characteristics (N = 148).

Variables	Categories	N (%)	Intention to care		
			M±SD	t/F	*p*
**Gender**	Female	140(94.6)	1.03±1.60	0.30	.762
	Male	8(5.4)	1.21±1.17		
**Age (in years)**	<30^a^	76(51.4)	1.06±1.54	5.66	.001
	30-39^b^	37(25.0)	0.41±1.50		(a,b<d)*
	40-49^c^	23(15.5)	1.29±1.60		
	≥50^d^	12(8.1)	2.42±1.09		
**Religion**	Follow	51(34.5)	1.29±1.59	-1.40	.165
	Do not follow	97(65.5)	0.91±1.57		
**Marital status**	Partnered	51(34.5)	1.18±1.59	0.75	.457
	Single	97(65.5)	0.97±1.58		
**Living together with family members**	Yes	131(88.6)	1.11±1.54	1.43	.115
	No	17(11.4)	0.53±1.82		
**Children**	Have	45(30.4)	1.4±1.60	-1.83	.069
	Do not have	103(69.6)	0.89±1.55		
**Education level**	3yr college ^a^	47(31.8)	0.60±1.68	5.73	.004
	Bachelors ^b^	94(63.5)	1.15±1.49		(a,b<c)*
	Master’s or higher ^c^	7(5.3)	2.57±0.74		
**Duration of job experience as a nurse (years)**	<1	9(6.1)	1.59±1.22	0.84	.476
	1–3	47(31.8)	1.18±1.51		
	4–6	25(16.9)	0.73±1.59		
	≥7	67(45.3)	0.99±1.66		
**Job position**	General-beside nurse ^a^	135(91.2)	0.93±1.53	4.36	.015
	Supervising nurse ^b^	6(4.1)	1.83±2.40		(a<c)*
	Head nurse ^c^	7(4.7)	2.52±0.60		
**Department**	Ward ^a^	127(86.5)	1.14±1.52	6.23	.003
	Intensive care unit ^b^	14(9.5)	-0.25±1.66		(a,c>b)*
	Others^†^ ^c^	6(4.0)	1.89±1.39		
**Type of work**	Three 8-hour shift pattern	135(91.2)	0.97±1.55	2.44	.091
	Fixed day or evening	5(3.4)	1.27±2.49		
	Day-time job	8(5.4)	2.21±1.05		
**Health status**	Healthy	67(45.3)	1.27±1.62	1.85	.160
	Moderate	68(45.9)	0.34±1.47		
	Unhealthy	13(8.8)	0.42±1.82		

Abbreviation: M = mean; SD = standard deviation.

*Scheffé test;

^†^Others included operating room, out-patients department, etc.

Based on the characteristics of participants’ clinical experience, 89.9% had received COVID-19-related training. Furthermore, 37.2% of the participants had previous experience of caring for patients infected with novel infectious diseases such as severe acute respiratory syndrome (SARS) and Middle East respiratory syndrome (MERS), and 25.7% had experience caring for patients with severe respiratory symptoms. In addition, 30.4% had experience working in an intensive care unit (ICU), and 20.9% had experience working in an emergency room (ER). Finally, 54.1% of the participants indicated that their clinical experience and skills were sufficient to care for COVID-19 patients (Table 2).

**Table 2:** Characteristics of participants’ clinical experience and differences in intention to care based on their clinical experience (N = 148).

Variables	Categories	N (%)	Intention to care		
			M±SD	t/F	*p*
COVID-19-related training	Yes	133(89.9)	1.10±1.58	1.38	.170
	No	15(10.1)	0.51±1.56		
Experience caring for patients infected with NID	Experienced	55(37.2)	1.28±1.37	1.42	.158
	Not experienced	93(62.8)	0.90±1.68		
Experience caring for patients with SRS	Experienced	38(25.7)	1.00±1.66	-0.19	.847
	Not experienced	110(74.3)	1.06±1.56		
Experience working in an ICU	Experienced	45(30.4)	1.00±1.58	-0.20	.843
	Not experienced	103(69.6)	1.06±1.59		
Experience working in an ER	Experienced	31(20.9)	1.41±1.31	1.46	.148
	Not experienced	117(79.1)	0.95±1.64		
Belief that their clinical experience and skills were sufficient to care for COVID-19 patients	Sufficient	80(54.1)	1.33±1.33	2.36	.020
	Not sufficient	68(45.9)	0.71±1.79		

Abbreviations: M = mean; SD = standard deviation; NID = novel infectious diseases; SRS = severe respiratory symptoms; ICU = intensive care unit; ER = emergency room.

### Level of positive psychological capital, nursing professionalism, and intention to care

The level of positive psychological capital of the study participants was 3.98±0.57. The level of nursing professionalism was 3.20±0.28, and level of intention to care was 1.04±1.58 (Table 3).

**Table 3:** Level of positive psychological capital, nursing professionalism, and intention to care (N = 148).

Variables	Range	M±SD	Min	Max
PPC	1–6	3.98±0.57	1.71	5.54
NP	1–5	3.20±0.28	2.52	4.12
Intention to care	-3–3	1.04±1.58	-3	3

Abbreviations: PPC = positive psychological capital; NP = nursing professionalism; M = mean; SD = standard deviation.

### Differences in intention to care by participants’ characteristics

Statistically significant differences were observed in nurses’ intention to care based on the following general characteristics of participants: age (F = 5.66, *p* = .001), education level (F = 5.73, *p* = .004), job position (F = 4.36, *p* = .015), and department (F = 6.23, *p* = .003). Specifically, participants aged more than 50 years (2.42±1.09) and those with an education level higher than a master’s degree (2.57±0.74) demonstrated the highest level of intention to care. In terms of job position, head nurses (2.52±0.60) demonstrated the highest level of intention to care, while in terms of department, those working in the ICU (-0.25±1.66) demonstrated the lowest level of intention to care (Table 1).

### Difference in intention to care by characteristics of participants’ clinical experience

The level of intention to care was significantly higher in participants who believed their clinical experience and skills were sufficient to care for COVID-19 patients (1.33±1.33) compared to when they did not (0.71±1.79) (t = 2.36, *p* = .020) (Table 2).

### Correlation between positive psychological capital, nursing professionalism, and intention to care

A statistically significant correlation was found between intention to care and both positive psychological capital (r = 0.30, *p* < .001) and nursing professionalism (r = 0.17, *p* = .041) (Table 4).

**Table 4:** Correlation among positive psychological capital, nursing professionalism, and intention to care (N = 148).

Variables	PPC	NP	Intention to care
	r(*p*)	r(*p*)	r(*p*)
PPC	1		
NP	.48(.001)	1	
Intention to care	.30 (< .001)	.17(.041)	1

Abbreviations: PPC = positive psychological capital; NP = nursing professionalism.

### Factors influencing intention to care

To identify the factors influencing nurses’ intention to care, a stepwise multiple regression analysis using the stepwise selection method was performed on factors that demonstrated a difference in intention to care—age, education level, job position, department, and belief that their clinical experience and skills were sufficient to care for COVID-19 patients—along with positive psychological capital and nursing professionalism, which correlated with intention to care. Nurses’ age, education level, job position, department, and belief that their clinical experience and skills were sufficient to care for COVID-19 patients were converted into dummy variables and included in the regression equation. To determine the presence of multicollinearity among independent variables, the basic assumption for regression analyses, the Durbin-Watson statistic and tolerance, and variance inflation factor (VIF) were calculated. The Durbin-Watson statistic was 2.185, close enough to 2 to confirm the absence of autocorrelation. Further, tolerance was 0.78–0.97, more than 0.1, and VIF was 1.072–2.352, less than 10, indicating that the issue of multicollinearity was not relevant to any of the variables. Moreover, the linearity and equal variance of the model was observed through residual analysis and the residuals were standardized to assume the normal distribution of the error terms, which indicated there was no value greater than the absolute value of 3 [25].

Factors influencing intention to care were identified as: nurses’ age (30<: β = .18, *p* = .026; ≥50: β = .23, *p* = .005), department (ICU: β = -.26, *p* = .001), belief that their clinical experience and skills were sufficient to care for COVID-19 patients (sufficient: β = .18, *p* = .019), and positive psychological capital (β = .22, *p* = .044). The explanatory power of the model (R^2^) was 48.0% (Table 5).

**Table 5:** Factors influencing intention to care (N = 148).

Variables		B	SE	β	t	*p*
(Constant)		-1.94	0.96		-2.03	.044
Age	<30	0.57	0.25	.18	2.24	.026
	≥50	1.32	0.47	.23	2.83	.005
Department	ICU	-1.39	0.40	-.26	-3.46	.001
Belief that their clinical experience and skills were sufficient to care for COVID-19 patients	Sufficient	0.56	0.24	.18	2.37	.019
PPC		0.61	0.23	.22	2.61	.044

R^2^ = .480, Adj. R^2^ = .231, F = 8.51.

*p* < .001, Durbin-Watson = 2.185.

Abbreviations: SE = Standard error; ICU = Intensive care unit; PPC = positive psychological capital.

Note: Age (reference: 30–39 years), educational level (reference: 3yrs college), job position (reference: general-duty nurse), department (reference: ward), subjective judgment that clinical experience and skills are sufficient to care for COVID-19 patients (reference: not sufficient), nursing professionalism, and PPC were included.

## Discussion

Based on the results of this study, participating nurses’ intention to care for COVID-19 patients was 1.04 (±1.58). The intention to care observed in the participants of this study was higher than that observed in previous studies that used the same tools to report nurses’ intention to care for patients with novel infectious diseases, regardless of prior experience [9, 18]. These findings reveal that nurses’ intention to care for patients infected with a novel infectious disease in a situation where a new infectious disease is prevalent, is higher than in normal situations that do not involve an outbreak. This may be attributed to the fact that during the COVID-19 pandemic, the severity of the situation was recognized at the national and global levels and shared widely through various platforms such as the media and SNS. In addition, support acknowledging the efforts of clinicians to overcome the situation was also extended. Furthermore, given that the data collection period of this study was prior to November 2020 when the number of cases surged in Korea—and the region in which data was collected had a lower of number cases than other regions, meant that nurses’ workload and stress levels were not as high; these may have influenced the study findings. A follow-up study to evaluate the appropriate number of patients with a novel infectious disease that should be assigned to each nurse considering their workloads, may be worthwhile.

Positive psychological capital is a concept derived from positive psychology and refers to individuals’ positive psychological state [26]. This positive psychological state is an emerging paradigm for human resource development that is characteristically manageable with the potential for development and improvement [12, 26].

According to the findings of this study, the level of positive psychological capital of the nurses caring for COVID-19 patients was higher than that of nurses with a relatively shorter clinical experience ranging from 13 to 36 months [27]. Similarly, the positive psychological capital of participants was higher than or similar to that of nurses in general hospitals and small- to medium-sized hospitals [28, 29]. These findings indicate that the level of positive psychological capital of nurses experiencing fear, anxiety, depression, and exhaustion in the COVID-19 pandemic are similar to normal or higher than normal levels. Positive psychological capital is a positive psychological state in which an individual pursues personal development, and that enables optimism and increased coping ability, while experiencing decreasing job-related burdens in challenging situations [30, 31]. Furthermore, positive psychological capital enhances life satisfaction through positive changes in individuals’ attitudes and behaviors toward their jobs and improves organization performance by inducing organizational change [30]. Amid the medical crisis of the COVID-19 pandemic, the utilization of nurses’ positive psychological capital should be actively sought as a personal and organizational coping strategy.

This study identified positive psychological capital as a factor that positively affects the intention to care. A direct comparison of the findings was not possible because of the lack of previous research on the influence of positive psychological capital on nurses’ intention to care in a crisis such as COVID-19. However, a systematic review examining the factors related to positive psychological capital among clinical nurses by Lee et al. [13] reported a negative correlation between positive psychological capital and turnover intention, a finding similar to that in this study. Positive psychological capital not only has a positive influence on the relationship between nurses and patients in the clinical setting [32], but also helps to maintain nurses’ physical, mental, and social well-being [33], while improving the quality of nursing care and organizational performance [30, 32]. The use of positive psychological capital to help nurses overcome fear, anxiety, job stress, depression, social isolation, and exhaustion in the COVID-19 pandemic should be explored. Previous research has framed positive psychological capital as a capacity that can be developed and improved through retrospective training and learning [34]. A follow-up study should be conducted to develop and test a program designed to increase nurses’ positive psychological capital, and an implementation strategy for its use during a medical crisis should be explored.

According to the findings of this study, subjective judgment that clinical experience and skills are sufficient to care for COVID-19 patients was identified as a factor influencing nurses’ intention to care. A direct comparison of results was not feasible as no study has yet explored the influence of this subjective judgment on nurses’ intention to care in a crisis such as the COVID-19 pandemic. Results for this study can be interpreted in the same ways as the findings from Ko et al. [35], which suggested the need for continuing education to induce intention to care in SRAS patients, have previous emergency and disaster experience [36], findings from previous studies that report nurses’ increased intention to respond if they have previous experience in caring for patients with infectious diseases [37], and previous studies that report relationship of confidence in personal skills with willingness to work [38]. This indicates that providing an opportunity for nurses to gain sufficient knowledge and experience regarding novel infectious diseases can promote their participation in a pandemic or related crisis. Thus, it will be essential to provide information and training on novel infectious diseases in preparation for future pandemics and for such efforts to be made at the government level as well as in the medical and nursing fields.

The level of nursing professionalism among nurses caring for COVID-19 patients in this study was lower than that reported in previous research on nurses caring for patients with a novel infectious disease in a non-pandemic situation [9, 18], as well as nurses working in general hospitals under normal circumstances [39]. Such results indicate that the level of nursing professionalism decreases in medical crises such as the COVID-19 pandemic. From a fundamental perspective of nursing as a profession, caring for patients is considered a nurse’s duty [9]. However, during an infectious disease outbreak, clinicians may face conflict between their safety as individuals and their duty to care as clinicians [40]. Nursing professionalism may be utilized as a strategy to overcome this ethical conflict.

In this study, nursing professionalism was not identified as a factor influencing nurses’ intention to care. This supports the findings of previous studies examining the factors influencing nurses’ intention to care for patients with a novel infectious disease and Ebola patients [9, 16, 18]. Nevertheless, findings from another study that presented factors that influence nurses’ intention to care for MERS patients suggested otherwise [17]. Further research is necessary to explore the correlation between nurses’ intention to care and nursing professionalism in a medical crisis.

This study is valuable in that it reviewed the factors influencing intention to care among bedside nurses providing direct care for COVID-19 patients during a pandemic. However, the study has a few limitations that should be considered when interpreting its findings. First, this study did not assess factors, such as fear, anxiety, job stress, depression, and burn out, in nurses who provided care for COVID-19 patients. Therefore, we could not interpret the influence these factors on the nurses’ intention to care for COVID-19 patients. Future studies that include and explore these factors should be conducted. Second, the participants of the study were nurses caring for COVID-19 patients randomly sampled from hospitals in regions with a relatively lower number of COVID-19 cases, which may present a challenge in generalizing the findings. Third, this study examined nurses’ intention to care for COVID-19 patients in a period during which COVID-19 response had gained the attention of the Korean nation and media. Thus, the potential for bias from social desirability in participants’ responses cannot be excluded and should be considered while interpreting the findings. Fourth, the data for the study were collected prior to November 2020 and thus did not reflect the situation after November 2020 when the number of cases surged. This should also be considered when interpreting the findings of this study. A future study examining nurses’ intention to care organized by time periods according to the number of cases may be beneficial. Finally, the reliability of the nursing professionalism questionnaire used in this study was low: Cronbach’s α = .63. This should also be considered when interpreting the findings.

## Conclusion

The results identified positive psychological capital as a factor influencing nurses’ intention to care for COVID-19 patients. Thus, the development of various programs to improve nurses’ positive psychological capital and follow-up studies analyzing their effects are needed. Furthermore, the subjective judgment that clinical experience and skills are sufficient to care for COVID-19 patients was also identified as a factor influencing intention to care. Specifically, it will be necessary to provide information and training regarding novel infectious diseases; this will enable nurses to prepare for future novel infectious disease pandemics through efforts at the government level and in the medical and nursing fields. Nursing professionalism was not identified as a factor influencing nurses’ intention to care for COVID-19 patients. We hope that future studies will be conducted to assess the association between the nurses’ intention to care and their professionalism.

## Supporting information

S1 Appendix Questionnaire (Korean version). (PDF)

S2 Appendix Questionnaire data. (XLSX)
