# Supplementary material for: Factors influencing nurses’ intention to care for patients with COVID-19: Focusing on positive psychological capital and nursing professionalism
Source: PLoS One. 2022 Jan 19;17(1):e0262786. doi: 10.1371/journal.pone.0262786 (PMC8769348; doi:10.1371/journal.pone.0262786)
Supplement: S1 Appendix — (PDF) [file pone.0262786.s001.pdf]

S1. Appendix Questionnaire

안녕하십니까?

본 설문 조사에 응해 주셔서 감사합니다.

본 연구는 간호사의 코로나바이러스감염증-19(COVID-19) 환자 간호의도에 영향을 미치는 요인을 파악하기 위한 것입니다. 본 연구결과는 COVID-19 환자를 간호하는 간호사들의 간호의도를 증진시키기 위한 중재 프로그램 개발의 기초자료가 될 것입니다

설문지를 작성하는데 총 10~15분 정도 소요됩니다. 귀하께서 응답하신 내용은 오직 연구 목적 외에는 절대로 사용하지 않을 것이며, 모든 자료는 비밀보장을 약속합니다. 또한 언제든지 연구 참여를 중단 할 수 있습니다.

귀하의 응답은 연구에 소중하게 사용될 것으로 각 문항에 솔직하게 빠짐없이 응답해 주시기를 부탁드립니다. 설문에 대하여 문의사항이 있으실 경우에 아래의 연락처로 연락 주시기 바랍니다.

바쁘신 시간에도 불구하고 설문조사에 소중한 시간을 할애해 주셔서 감사드립니다.

2020년 8월

- 연구자: 조선대학교 대학원 간호학과 정선아
- 연락처: 010-2075-2074
- E-mail: sunaj08@naver.com
  jsa122@csuh.co.kr

※ 귀하는 코로나바이러스감염증-19(COVID-19) 환자를 직접 간호한 경험이 있습니까?
① 없다(⇒ 대상자 아님) ② 있다 (⇒ 설문을 진행하여 주십시오)

Ⅰ. 다음은 긍정심리자본을 측정하기 위한 것입니다. 평소 귀하 자신에 대해 어떻게 생각하는지를 알아보고자 하는 항목입니다. 귀하의 의견에 가까운 곳에 √표 해주시기 바랍니다.

번호	문항	전혀 그렇지 않다	그렇지 않다	별로 그렇지 않다	조금 그렇다	그렇다	매우 그렇다
1	나는 장기적 문제를 분석해서 해결책을 찾는 일에 자신이 있다.						
2	나는 상사들과의 회의(예:부서회의, 집담회 등)에서 내 업무 분야를 대표해 말하는 것에 자신이 있다.						
3	우리 조직의 전략에 관하여 논의할 때 기여할 자신이 있다.						
4	나는 나의 업무분야에서 목표설정을 도울 수 있는 자신감이 있다.						
5	나는 외부사람(예:타부서, 타 기관, 환자)들과 현안을 논의하는 일에 자신이 있다.						
6	나는 동료들에게 필요한 정보를 발표하는데 자신이 있다.						
7	나는 업무 상 난관에 부딪혔을 때, 이를 해결할 수 있는 여러 방법들을 생각해 낼 수 있다.						
8	현재, 나는 업무상의 내 목표를 열성적으로 추구하고 있다.						
9	어떤 문제라도 해결할 수 있는 방법들은 다양하다.						
10	현재, 나는 업무적으로 꽤 성공했다고 스스로 평가한다.						

11	나의 업무 목표를 달성하기 위한 다양한 방법들을 생각해 낼 수 있다.						
12	현재, 나는 업무 측면에서 스스로 설정한 목표를 달성하고 있다.						
13	나는 직장에서 좌절을 겪었을 때, 이를 떨치고 회복하기가 어렵다.						
14	나는 보통 직장에서 겪게 되는 어려움을 여러 가지 방법으로 잘 대처한다.						
15	나는 업무 중에 필요하다면 혼자 힘으로 일을 처리할 수 있다.						
16	나는 직장에서 스트레스를 주는 일들을 보통 잘 이겨낸다.						
17	나는 과거에 어려움을 겪었던 경험이 있기 때문에, 직장에서의 힘든 시간을 잘 극복해 낼 수 있다.						
18	나는 현재 업무와 관련된 여러 일을 동시에 잘 처리할 수 있다.						
19	나는 업무적으로 불확실한 상황에서도 대체로 좋은 결과가 나올 것을 기대한다.						
20	업무적으로 문제가 생길 것 같은 경우, 실제로도 문제가 생긴다.						
21	나는 항상 내가 맡고 있는 일의 긍정적인 면을 보려고 한다.						
22	나는 내가 하는 일에 대해 낙관적인 희망을 갖고 있다.						
23	내가 맡고 있는 일들은 결코 내가 원하는 방식으로 풀리지 않는다.						
24	나는 힘든 일이 있으면 좋은 일도 있을 것이라는 믿음으로 일한다.						

Ⅱ. 다음은 간호사로서 간호전문직관을 측정하기 위한 것입니다. 평소 귀하의 느낌에 가장 가까운 곳에 √표 해주시기 바랍니다.

번호	문항	항상 그렇다	대체로 그렇다	보통이다	대체로 그렇지 않다	절대 그렇지 않다
1	나는 정기적으로 (간호) 전공 잡지를 읽는다.					
2	다른 전문직이 우리(간호직)보다 실제적으로 사회에서 더 중요하다.					
3	내 (간호)업무에서 무엇을 해야 할지는 내가 스스로 결정한다.					
4	나는 지역의 전문가(간호사) 모임에 정기적으로 참석한다.					
5	나는 우리 직종(간호직)이 다른 어떤 전문직보다 사회에서 없어서는 안된다고 생각한다.					
6	내 동료들은 서로의 업무수행능력에 대해 잘 알고 있다.					
7	우리 (간호)전문직에 종사하는 사람들은 일에 대한 참 소명의식을 가지고 있다.					
8	내 (간호직의)전문성에 대한 중요성이 때로는 지나칠 정도로 강조되기도 한다.					
9	이 분야(간호직)에서 사람들이 헌신하는 것은 매우 보람 된 것이다.					
10	내 스스로의 판단을 실행에 옮길 기회를 자주 갖지 못한 편이다.					
11	나는 (간호)전문직 단체에 회원으로 가입해야 한다고 생각한다.					
12	몇 몇 다른 직업은 내 직업(간호직)보다 사회에서 더욱 중요하다.					
13	우리 전문직의 문제점은 동료가 무엇을 하는지 실제적으로 서로 잘 모른다는 것이다.					
14	우리 분야 종사자들이 높은 이상적 태도를 유지하는 것은 바람직하다.					

15	(간호)전문직 단체는 일반회원들을 위해 충분한 역할을 감당하지 못하고 있다.					
16	우리는 서로의 (간호)수행능력을 판단할 방법이 없다.					
17	나는 (간호)전공 잡지를 읽으려 하지만, 실제적으로는 자주 읽지 못한다.					
18	수입이 감소되더라도 대부분은 이 직종(간호직)에 계속 종사할 것이다.					
19	내 의사결정은 검토를 받아야 한다.					
20	동료들이 어떻게 일을 하는지 평가할 기회가 별로 없다.					
21	(간호)업무와 관련된 거의 모든 문제는 나 스스로 해결한다.					
22	세상에 꼭 필요한 직업이 있다면, 그것은 우리 직업(간호직)이다.					
23	내 동료들은 서로가 일을 잘 하고 있는지 비교적 잘 파악하고 있다.					
24	(간호사들 중에) 자신의 일에 대한 신념이 없는 사람은 거의 없다.					
25	내가 내린 결정의 대부분은 다른 사람이 다시 검토한다.					

Ⅲ. 다음은 코로나바이러스감염증-19 환자 간호의도에 대한 문항입니다. 평소 귀하의 느낌에 가장 가까운 곳에 √표 해주시기 바랍니다.

번호	항 목	전혀 그렇지 않다			<---------->			매우 그렇다
1	나는 코로나바이러스감염증-19 환자의 담당 간호사가 된다면 나는 기꺼이 간호에 참여하겠다.							
2	나는 코로나바이러스감염증-19 환자를 간호할 마음이 있다.							
3	나는 코로나바이러스감염증-19 환자 간호에 자발적으로 참여하겠다.							

IV. 다음은 귀하의 일반적 특성에 대한 질문입니다. 해당 란에 √표 또는 직접 적어 주십시오.

1. 귀하의 성별은 어떻게 되십니까? ① 남자 ② 여자

2. 연령: 실제 출생년도를 적어 주십시오. (__________년)

3. 종교가 있으십니까?
   ① 없다 ② 있다 (⇒ 3-1. 있으시면 해당 종교에 표시하여 주십시오)

3.1 ① 기독교 ② 불교 ③ 천주교 ④ 기타 (   )

4. 귀하는 배우자가 있습니까?
   ① 있음 ② 없음

5. 동거하는 가족의 형태는 어떠합니까?
   ① 본인+부모님 ② 본인+부모님+형제/자매 ③ 본인+배우자
   ④ 본인+배우자+자녀 ⑤ 본인+배우자+자녀+시부모(또는 친정부모)
   ⑥ 본인+형제/자매 ⑦ 혼자 거주 ⑧ 본인+친구 ⑨ 기타 (   )

6. 자녀는 몇 명인가요?
   ① 0명 ② 1명 ③ 2명 ④ 3명 ⑤ 4명 이상

7. 귀하의 최종학력은 어떻게 되십니까?
   ① 전문학사 ② 학사(석사 과정 중 포함) ③ 석사(박사 과정 중 포함) ④ 박사

8. 귀하는 현재 병원에서의 직위는 어떻게 되십니까?
   ① 일반간호사 ② 주임(책임)간호사 ③ 수간호사 이상 ④ 전문간호사 ⑤ 기타(   )

9. 현재 귀하의 근무부서는 어디입니까?
   ① 병동 ② 중환자실 ③ 응급실 ④ 외래 ⑤ 기타(   )

10. 귀하의 임상경력은 어떻게 되십니까? ____년_____개월

11. 중환자실 근무 경험이 있으십니까?
   ① 있다 ② 없다

12. 응급실 근무 경험이 있으십니까?
① 있다 ② 없다

13. 현재 귀하의 근무형태는 어떻게 되십니까?
① 3교대 ② Day/Evening 고정 ③ Night 고정 ④ 상근 ⑤ 기타(   )

14. 현재 귀하의 고용형태는 어떻게 되십니까?
① 정규직 ② 계약직 ③ 기타(   )

15. 귀하의 현재 건강상태는 어떻다고 생각하십니까?
① 건강한 편이다 ② 보통이다 ③ 건강하지 못한 편이다

V. 다음은 귀하의 직업경험 특성에 대한 질문입니다. 해당 란에 √표 또는 직접 적어 주십시오.

1. 귀하는 COVID-19 또는 신종감염병 관련 교육을 받으신 적이 있습니까?
① 있다 ② 없다

2. 귀하는 개인 보호용구(예: N95마스크, Level D이상의 전신 보호복, PAPR 등) 착탈의 방법에 대해 교육을 받으신 적이 있습니까?
① 있다 ② 없다

3. 귀하는 이전에 신종감염병(예: SARS, MERS 등) 환자(의심환자 포함) 간호경험이 있으십니까?
① 있다 ② 없다

4. 귀하는 이전에 중환자실에서 중증 호흡기 환자를 간호한 경험이 있으십니까?
① 있다 ② 없다

5. 귀하는 본인이 COVID-19 환자를 간호하기에 충분한 임상적 경험과 기술이 있다고 생각하십니까?
① 그렇다 ② 그렇지 않다

- 설문에 참여해 주셔서 감사합니다. -
